# Operationalization, implementation, and evaluation of Collaboration Planning: A pilot interventional study of nascent translational teams

**DOI:** 10.1017/cts.2020.515

**Published:** 2020-07-24

**Authors:** Betsy Rolland, Linda Scholl, Sainath Suryanarayanan, Peggy Hatfield, Kate Judge, Christine Sorkness, Elizabeth Burnside, Allan R. Brasier

**Affiliations:** 1 Institute for Clinical and Translational Research, School of Medicine and Public Health, University of Wisconsin-Madison, Madison, WI, USA; 2 Carbone Cancer Center, School of Medicine and Public Health, University of Wisconsin-Madison, Madison, WI, USA; 3 Mayo Clinic College of Medicine and Science, Rochester, MN, USA; 4 Nelson Institute for Environmental Studies, Center for Culture, History and Environment, University of Wisconsin-Madison, Madison, WI, USA; 5 Department of Radiology, School of Medicine and Public Health, University of Wisconsin-Madison, Madison, WI, USA; 6 Department of Internal Medicine, School of Medicine and Public Health, University of Wisconsin-Madison, Madison, WI, USA

**Keywords:** Team science, collaboration, collaboration planning, education, workforce development

## Abstract

**Background:**

The University of Wisconsin Institute for Clinical and Translational Research hub supports multiple pilot award programs that engage cross-disciplinary Translational Teams. To support those teams, our Team Science group aims to offer a learning experience that is accessible, active, and actionable. We identified Collaboration Planning as a high-impact intervention to stimulate team-building activities that provide Translational Team members with the skills to lead and participate in high-impact teams.

**Methods:**

We adapted the published materials on Collaboration Planning to develop a 90-minute facilitated intervention with questions in 10 areas, presuming no previous knowledge of Science of Team Science (SciTS) or team-science best practices. Attendees received a short follow-up survey and submitted a written collaboration plan with their first quarterly progress report.

**Results:**

Thirty-nine participants from 13 pilot teams from a wide range of disciplines engaged in these sessions. We found that teams struggled to know who to invite, that some of our questions were confusing and too grounded in the language of SciTS, and groups lacked plans for managing their information and communications. We identified several areas for improvement including ensuring that the process is flexible to meet the needs of different teams, continuing to evolve the questions so they resonate with teams, and the need to provide resources for areas where teams needed additional guidance, including information and data management, authorship policies, and conflict management.

**Conclusions:**

With further development and testing, Collaboration Planning has the potential to support Translational Teams in developing strong team dynamics and team functioning.

## Introduction

Clinical and translational science is inherently team-based, requiring collaborators with different skill sets and expertise to work together across the translational spectrum. The most recent Clinical and Translational Science Award (CTSA) Request for Applications included a focus on providing Translational Teams with team-science education and support. Translational Teams are a special case of cross-disciplinary team focused on addressing unmet health needs, with a dynamic and diverse membership whose goal is to advance a product (device/drug/diagnostic), behavioral intervention, or evidence-based approach toward sustainable improvements in human health [1,2]. At the University of Wisconsin-Madison Institute for Clinical and Translation Research (UW-ICTR), we are building infrastructure to support Translational Teams by developing interventions for both existing and nascent teams that provide team-science education that is accessible, active, and actionable. *Accessible* learning does not require substantial pre-existing knowledge in order to be effective. Here, we designed an intervention that does not require knowledge of the principles of team science. *Active* learning engages individuals and teams as participants in their learning, teaching skills, as opposed to passive, didactic delivery of information. Finally, *actionable* learning provides a roadmap for taking what the team has learned and using it to address the strengths and weaknesses of the team, with priorities for improvement. In short, the team should walk away with a deeper knowledge of how their team processes impact their scientific work; the skills to design, assess, and, when needed, correct their team processes based on the Science of Team Science (SciTS) evidence base; and the attitude that team processes are something they can impact.

We identified Collaboration Planning as a high-impact intervention to stimulate team-building activities that provide Translational Team members with the skills they need to lead and participate in high-impact teams. The goal of this intervention is to help teams develop evidence-informed or evidence-based collaborative processes before embarking on their project and to focus attention on team processes as an important contributor to the overall success of the project. By talking about potentially contentious issues at the beginning of the project and anticipating potential areas of conflict before they happen, Translational Teams can build trust among members and proactively impact their team dynamics and team functioning [3].

The Collaboration Planning approach was developed by a group of SciTS researchers based at the National Cancer Institute and the National Science Foundation. First published as a white paper in 2014 [4], then as a poster at the SciTS conference in 2015 [5], the idea behind Collaboration Planning is straightforward: teams benefit from advance discussion of known, potential challenges to collaborative research. The authors reviewed the existing SciTS literature, which draws from the fields of organizational psychology, management theory, teams research, sociology, psychology, and others, and identified 10 areas of focus for team discussion: 1. Rationale for Team Approach and Configuration; 2. Collaboration Readiness; 3. Technological Readiness; 4. Team Functioning; 5. Communication and Coordination; 6. Leadership, Management, and Administration; 7. Conflict Prevention and Management; 8. Training; 9. Quality Improvement Activities; 10. Budget and Resource Allocation (Table 1). The whitepaper and poster describe each area of focus, the rationale for selection, and relationship to effective team science. (Note: After our pilot was completed, the authors of the whitepaper and poster published an update to their framework [6].) While the plans that emerge from these discussions are important in their own right, the act of having those discussions as a team can also help build the trust and positive relationships that are so critical to successful teams. Furthermore, by using the Collaboration Planning intervention to implement SciTS-supported team best practices, teams also can improve their reproducibility behaviors [4]. To our knowledge, Collaboration Planning as an intervention to train Translational Teams has not been operationalized, implemented, and evaluated.

**Table 1.** Summary of collaboration planning’s 10 areas of focus [1,2]

Rationale for team approach and configuration	Why is this work being done as a team?
Collaboration readiness	How ready are the individuals, team(s), and institution(s) to collaborate?
Technological readiness	What technologies will be used to support collaboration?
Team functioning	What team processes will be leveraged to support collaboration?
Communication and coordination	How will the team communicate and coordinate their work?
Leadership, management, and administration	What approach will the team take to leadership, management, and administration of the project?
Conflict prevention and management	How will the team prevent and manage potential conflicts?
Training	How will team members be trained to collaborate on this project?
Quality improvement activities	How will the team assess the quality of its team processes?
Budget and resource allocation	How will the team use its resources to support strong team processes?

Here, we share how we adapted the published Collaboration Planning framework into a standardized direct intervention for, primarily, nascent teams, report the results of our follow-up survey, observations, and analysis of the resulting Collaboration Plans, then discuss what we learned, and describe future work.

## Methods

The UW-ICTR Pilot Awards program aims to catalyze interdisciplinary research that spans the translational research spectrum from pre-clinical to public health. The program solicits applications yearly through seven distinct requests for applications (RFAs). Funded projects span this translational research spectrum; the composition of their associated teams includes academic, clinical, and community members. To learn how to assist teams in achieving their project outcomes, we invited teams awarded in 2018 to share feedback on core concepts in team science (dynamics, readiness to collaborate, communication, etc.). Overall, teams listed practical activities aimed at orienting and communicating expectations, understanding team dynamics, and decision-making as most useful. Given this feedback, we identified collaboration planning as a potential experience to achieve our goal of supporting teams. Thus, we offered it to all funded pilot projects in 2019, including pre-clinical, clinical, clinical implementation, and public health pilot research projects. For purposes of this discussion, we divide the seven pilot mechanisms into two groups: (1) pre-clinical projects and (2) community-engaged research projects (CEnR).

Starting with the work by Hall, Vogel, and Crowston on writing a collaboration plan [4,5] coupled with our own experience supporting, leading, and participating in collaborative team science [7–9], we adapted the materials to develop questions in each of the 10 areas of focus (Appendix A). The goal of the question format was to stimulate explicit discussion among the team members about each of the areas, rather than for the team members to have a definitive answer. These discussions would help them begin thinking about and planning for ways to improve their team processes. The UW-ICTR pilot award administrators (KJ and PH) then invited funded pilot awardees using a message crafted to incentivize senior researchers to participate by (a) stressing the usefulness of the session to early-stage investigators on their teams and (b) highlighting how this information could be used in future grant applications (Appendix B). We administered these questions in a 90-minute facilitated session with the pilot teams who accepted this invitation. Each session began with introductions, followed by a brief (3–5 min) overview of the project delivered by the principal investigator (PI), then a discussion with the team members covering the 10 areas of the worksheet. Each team meeting was attended by the PI(s) and key team members, a UW-ICTR facilitator, and a UW-ICTR evaluator. The role of the facilitator (BR) was to walk the team through the questions; explain the relevance of each question to the practice of team science, as necessary; keep the team on track and on time; serve as a resource for SciTS knowledge when questions came up; and raise important and otherwise unforeseen issues for which consideration, discussion, and planning would position the team for success. The role of the evaluator (LS or SS) was to observe the sessions, note any questions on the worksheet that did not seem to make sense to the team members, and make overarching observations about the direction of the conversation. The evaluator did not actively participate in the discussion. After each meeting, attendees received a short survey (Appendix C) soliciting feedback about their experience with collaboration planning, including the areas they thought themselves most and least likely to use, as well as whether they found the overall experience valuable. Team PIs were asked to submit a written Collaboration Plan with their first pilot award quarterly progress report, following the general format of the Collaboration Planning worksheet. These plans were analyzed for common themes (BR). We did not assess the process teams followed in creating the plans after our session, nor did we attempt to judge the “quality” of the plans, as we currently lack *a priori* criteria by which to do so. Teams have not received any feedback on their plans.

## Results

Twenty-six teams were invited to participate in the intervention; 13 accepted, with a total of 39 participants. Meetings were attended by an average of 3.3 participants and had a range of one to five participants. Six teams were representative of the pre-clinical projects, while seven teams were representative of the CEnR projects. Participants included scientists across the translational research spectrum, such as basic translational scientists, clinical investigators, population scientists, social and behavioral researchers, health services researchers, and community partners. Some sessions included only the team’s PI, while other sessions included the PI and all collaborators. Meeting times ranged from 30 to 90 minutes.

### Survey Results

**Fig. 1.**
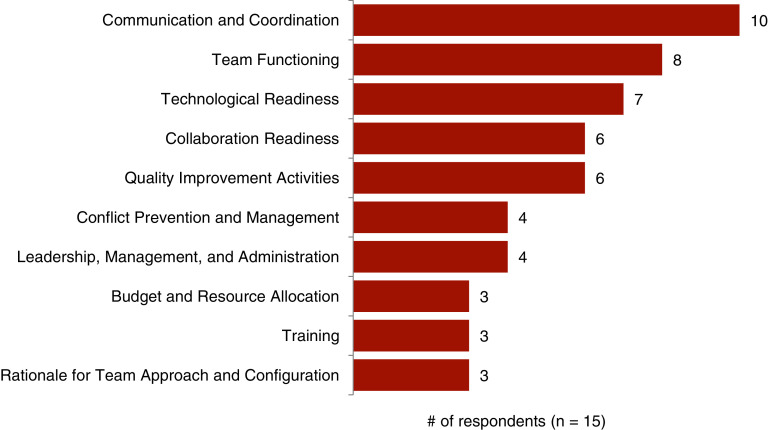
Areas of collaboration planning that respondents anticipated using the most in future collaborative work.

We received 15 individual responses to the follow-up survey from the 39 attendees who participated (38%), with 100% of respondents finding the exercise somewhat or very valuable. Respondents anticipated using the Communication and Coordination section the most (67%, *n* = 10), followed by Team Functioning (53%, *n* = 8), and Technological Readiness (47%, *n* = 7) (Fig. 1). The areas that teams reported they were least likely to use included the Rationale for Team Approach and Configuration (60%, *n* = 9), Budget and Resource Allocation (47%, *n* = 7), and Collaboration Readiness (47%, *n* = 7). In open-ended questions, respondents were asked for additional comments about what collaboration issue(s) they anticipated would be most problematic for their team and for any suggestions on improving the intervention. Of the 10 respondents who answered the question about anticipated problems, five reported they did not anticipate problems. Others commented on anticipated issues such as refining team logistics, tracking team activities, filling gaps when experienced collaborators left the team, and advancing quality improvement activities. Suggestions for improving the process included continuing to work with teams over the course of their pilot (i.e., beyond the initial collaboration planning exercise); providing additional concrete examples and resources for teams when they faced issues; focusing on encouraging all participants to speak up, especially graduate students and post-doctoral researchers; and targeting the collaboration planning to the team’s stage of development.

### Qualitative Session Observations

The qualitative observations yielded several key insights. After receiving the invitation to participate, team leaders were not always sure who to invite to the session. Research has shown that team boundaries can be fluid and who is “in” and who is “out” of the team is not always clear [10], so our request to invite “members of [their] team” to the session was not as straightforward as we had anticipated. Additionally, these teams were supported by modest funds ($50,000–$75,000), so many collaborators were either included on the project with minimal or no effort, and team leaders were reluctant to “spend” that effort on the Collaboration Planning. This challenge was especially apparent for PIs of community-engaged research teams, which often included busy community partners.

Participants, especially those on the pre-clinical science teams, sometimes struggled to understand questions like 4.b “How will your team create shared mental models/frameworks of your project’s scientific concepts?” With a little support from the facilitator, most were able to craft an answer, but we were clearly pushing them to think about their work in a way that they were not accustomed to doing, using language that was often unfamiliar.

We observed that none of the groups had explicit plans for how to manage their project information and their communications. Many were using role-defined file share (Box), university-hosted shared network drives, online shared documents (i.e., Google docs), electronic lab notebooks, email, texting, and phone calls to communicate and to store and share information, yet none had a policy or any guidelines for what types of communications should take place via which technology or how those communications should be stored or tracked. For example, one team used email and texting to make decisions about their work but did not document those decisions somewhere more permanent and accessible. Another team reported a lab technician that had documented several failed experiments in their personal lab notebook and then left the university, making that documentation inaccessible to the rest of the team. The final two areas of focus, Quality Improvement Activities and Budget and Resource Allocation, appeared to be confusing for all the teams. The intention of these sections is to think about how the *team processes* discussed in the previous eight sections will be supported. Despite instructions from the facilitator to focus on these team processes instead of their scientific work, teams talked about how they would think about the quality of their *scientific* work or allocate their *scientific* budgets. We were not able to find language that brought them back to team processes, despite trying different approaches over the course of our sessions.

### Observations from the Collaboration Plans

As of this writing, we have received written collaboration plans from 6 of the 13 teams. Two of these written plans were from the pre-clinical science teams, while the remaining four came from the CEnR teams. One pre-clinical science team submitted a very brief plan, including four one-sentence bullet points summarizing our discussion, but not covering any of the 10 areas of focus specifically. The remaining five plans covered the 10 areas of focus to differing degrees (Table 2). Three of the plans covered all 10 areas of focus, one covered seven of the areas, and one covered just five of the areas. The areas that were covered by all five of the teams that addressed specific areas of focus included: Technological Readiness (#3); Leadership, Management, and Administration (#6); Conflict Prevention and Management (#7); and Quality Improvement Activities (#9). Interestingly, these four areas were not those that respondents thought they were “most likely to use” or “least likely to use” in the future based on survey responses.

**Table 2.** Topics covered by team principal investigators in submitted collaboration plans

	CEnR1	Pre-clinical1	CEnR2	CEnR3	CEnR4	Pre-clinical2
1. Rationale for team approach and configuration	X	X		X	X	
2. Collaboration readiness	X	X		X	X	
3. Technological readiness	X	X	X	X	X	
4. Team functioning	X	X	X	X		
5. Communication and coordination	X	X		X	X	
6. Leadership, management, and administration	X	X	X	X	X	
7. Conflict prevention and management	X	X	X	X	X	
8. Training	X	X		X		
9. Quality improvement activities	X	X	X	X	X	
10. Budget and resource allocation	X	X		X		

## Discussion

This pilot test of a Collaboration Planning intervention surfaced many valuable lessons that we have applied to improve the process. First, the process must be highly flexible in order to meet teams and participants “where they are” in terms of characteristics like team maturity; the degree of team science and interdisciplinarity required; where the work sits on the translational spectrum; or the size of the team. One team was led by a new PI just starting a lab, while another was led by a PI leading his last project before retirement. The first PI required a deep dive into questions of lab management, while the second was more concerned with how to engage his community partners in a way that he could transition those critical collaborations to his junior colleague. One team was using an established protocol to collect data for a future grant proposal in a way that required very little collaboration between the two labs; this project hardly qualified as team science. Yet other teams were starting brand-new collaborations with colleagues they had never worked with before, necessitating much more delicate negotiations. Senior researchers often opened the meeting by telling us that they had been doing team science for decades and were only there to support their junior colleagues. Yet, those senior researchers who engaged in the intervention also found themselves taking notes on the conversation and thinking about changing long-standing team practices.

Second, the language we were using was still too grounded in the language of the SciTS and social science. Even the term itself, “team science,” was unclear and confusing. What exactly constituted team science? Was their team conducting team science? As we prepared for the second round of piloting our intervention (discussed in the Future Directions section below), we switched to language more focused on “collaborative science,” which is broader and seemed to resonate better with these teams.

Third, the pre-clinical science teams and the CEnR teams engaged with Collaboration Planning differently. Most of the pre-clinical science teams were conducting research that was primarily protocol-driven, in pursuit of data for a future grant application, requiring minimal interaction among collaborators or across labs. The CEnR teams, on the other hand, were engaged in more “sense-making” work that required more information sharing and more discussion as their topics were perhaps more ambiguous and open-ended than those of the pre-clinical science teams. As such, the pre-clinical science teams needed fewer systems to support their interactions, and some of the questions about conflict management and creating shared frameworks did not resonate as strongly with those teams. The CEnR teams, on the other hand, were already actively engaged in those challenges and were more receptive to tools and approaches that could support that aspect of their work.

Fourth, many of the questions in the session aim to surface and identify challenges in collaboration. Once the challenges had been identified, however, participants wanted solutions and guidance on how to navigate areas such as information and data management, authorship policies, and managing conflict. Drawing upon the resources of UW-ICTR and the UW more broadly, we are developing those resources (identified in Appendix D) for the next round of interventions. For example, we will be working with our UW-Madison Ebling Library for the Health Sciences to create tutorials on using electronic lab notebooks to share information in teams in a way that persists.

Fifth, participation by the entire team is essential, albeit challenging as described above. Decisions are made, relationships and trust are built during this session, and if members of the team are not included, it can be detrimental to the overall team dynamics. It is the facilitator’s job to ensure that all those present are participating in the conversation, but that is a challenging task, especially when the team involves graduate students and community partners who may be less confident in their status or the value of their contributions.

The impact of conducting Collaboration Planning with teams is difficult to measure. While participants reported that they found it valuable, the real test will be whether they take the lessons learned forward into the research collaborations. Will the Collaboration Plan that teams created after the session be one that they actually use if conflicts arise or new people join the team? Can it be used as a document that defines the team’s culture? Will explicit discussions about data retention and management improve methods reproducibility, as we have proposed in previous work [11]? These remain open questions that we hope to explore in future research.

## Limitations

This pilot study is limited in its generalizability by the small number of teams participating in this intervention, as well as the fact that teams self-selected to participate in the Collaboration Planning sessions. The teams that participated may not be representative of Translational Teams, in general, in that they were pilot teams with modest funding, and may not have been representative of all of the teams funded by these two pilot mechanisms. We also did not gather data on team characteristics beyond what we could glean from their applications to the pilot award mechanism.

## Future Directions

After the first round of pilot testing described here, we revised the worksheet substantially (Appendix D) and tested it with an additional seven nascent Translational Teams, all of whom had heard from colleagues or UW-ICTR program staff about the intervention and asked us to facilitate a session for them. As we work with more teams, we will continue to revise the questions to better serve our Translational Teams. We are also creating a facilitator’s guide that outlines team-science principles that serve as the foundation for the worksheet questions and can help the facilitator guide the conversation and respond to questions from participants. This facilitator’s guide will also enable us to scale Collaboration Planning as a UW-wide intervention by conducting train-the-trainer workshops, as well as disseminating it to the CTSA network.

While we did not investigate the process teams followed in writing their collaboration plan after our session, nor did we attempt to analyze the quality of the plans, we plan to develop approaches to do so in the future. The latter is an area for future research and must be tied to team outcomes in some way. One potential outcome is increased reproducibility of research. Our team has developed a rubric to assess team-based behaviors that may increase rigor and reproducibility [11], which has been identified as a challenge for translational science, and we plan to either extend that rubric to apply to Collaboration Plans or develop a complementary rubric to assess Collaboration Planning specifically.

Furthermore, we plan to collect longitudinal data about the outputs and outcomes of the teams that have gone through the Collaboration Planning intervention, perhaps even retrospectively comparing them to UW teams that did not. We will also continue to gather data about other, unanticipated ancillary impacts such as connections being made between teams and, say, the medical librarians who help with data management plans or UW-ICTR staff. As we develop plans to scale and disseminate, we will also be considering ways to test the intervention more rigorously to create the evidence base for the efficacy and effectiveness of the Collaboration Planning intervention so that teams can point to this evidence and include funding for the intervention in their grant proposals.

We are also exploring ways to help teams use these documents more fully. A Collaboration Plan can be used to onboard new team members, to help create a culture of collaboration, and even serve as a basic governance document for smaller collaborations. It should be revisited periodically during the life of a team to ensure it still represents the team’s approach to and ethos of collaboration.

## Conclusion

We believe that, with further development and testing, the structured process of Collaboration Planning, an exercise situated at the intersection of team-science education and team-science interventions, will provide an accessible, active, and actionable approach to improving a team’s functioning not just through its content but by virtue of the participation itself. The result of the Collaboration Planning intervention is not simply a document that is written and then placed on a shelf. It is a living document that should change and evolve as the team does, representing and reifying its values, and serving as a way to communicate the mission and goals of a Translational Team. A second, though equally important, result of collaboration planning is the critical relationship and trust building that takes place when teams discuss potentially sensitive topics in a psychologically safe space with a neutral facilitator. This latter impact of collaboration planning is especially difficult to measure, yet critically important for teams to understand. The resulting improvement in both team dynamics and team functioning can lead to improved scientific outcomes and high-impact clinical and translational science.

## Supplementary material

For supplementary material accompanying this paper visit https://doi.org/10.1017/cts.2020.515. click here to view supplementary material
