# Supplementary material for: Operationalization, implementation, and evaluation of Collaboration Planning: A pilot interventional study of nascent translational teams
Source: J Clin Transl Sci. 2020 Jul 24;5(1):e23. doi: 10.1017/cts.2020.515 (PMC8057480; doi:10.1017/cts.2020.515)
Supplement: Supplementary file 1 [file S2059866120005154sup001.docx]

**Appendix A**

**Collaboration Planning Worksheet (v2. July 18, 2019)**

**Adapted from *How to Write a Collaboration Plan*, Hall, Crowston, and Vogel [4, 5]**

1. Rationale for Team Approach and Configuration
   1. Why are you doing this work as a team?
   2. Who is on the team and why?
   3. What skill sets are missing from this team?
2. Collaboration Readiness (Individual and Team)
   1. Have members of the team worked on team science projects previously?
   2. Have members of the team worked together previously?
   3. Do members of the team come from closely-related disciplines?
   4. Have members of the team engaged in any training in team science prior to this meeting?
3. Technological Readiness
   1. What communication technologies (WebEx, email) will you use to work together?
   2. What coordination technologies (shared calendar, Box, shared drive, project management tools) will you use to work together?
   3. What data management and analysis tools will you use to work together?
   4. Are your individual systems interoperable?
4. Team Functioning
   1. How will your team create a shared vision of what success looks like for the project as a whole and the individual components?
   2. How will your team create shared mental models/frameworks of your project’s scientific concepts?
   3. Who will do what on your team and in collaboration with whom?
   4. How will your team hold one another accountable for delivering high-quality work on time and as agreed upon?
5. Communication & Coordination
   1. How often will your team meet and by what modality (in-person, WebEx, phone)?
   2. Are there sub-teams that will meet? If so, how will the outcomes of those meetings be communicated to the larger group?
   3. How will you track tasks and their completion?
   4. How will you communicate as a group (email listserv, Google groups)?
6. Leadership, Management, and Administration
   1. How will leadership be shared (ensuring vision is maintained, directing activities, tracking tasks and deliverables, overseeing communications, addressing conflict)?
   2. How will you encourage all team members to show leadership?
   3. How will tasks be identified, assigned, tracked, and judged complete?
   4. How will the priority of tasks be decided if there are conflicts?
7. Conflict Prevention and Management
   1. Do you anticipate publications or intellectual property arising from this project?
   2. If so, what will your authorship or attribution policies be?
   3. How will disputes over resources or deliverables be resolved?
   4. How will the leadership team ensure that all members are encouraged to participate and add their unique perspectives to the conversation?
8. Training
   1. Will the project require collaborators to cross-train on methods or tools?
   2. Will team members need training on how to use the communication, coordination, or data systems?
   3. What team-based skills will the team need, beyond what we’ve done today (e.g., conflict resolution, effective communication, information management, leadership)?
9. Quality Improvement Activities
   1. How will you assess the functioning of your team?
   2. How will you ensure continued improvement of the team function?
   3. How will you build in reflection time at each milestone?
10. Budget & Resource Allocation
    1. How will you allocate resources to support team function?
    2. Who will be responsible for ensuring strong team function?

**Appendix B**

**Email Invitation to Pilot Teams**

Dear [PIs],

As promised in your ICTR pilot award letter, I’m writing with details about an exciting team science opportunity for ICTR pilot awardees and their teams.  Specifically, we would like to invite you and your team to participate in a facilitated Collaboration Planning process.

**What is Collaboration Planning?** Designed by team-science experts at the NIH and NSF drawing upon decades of research on teams and collaboration, collaboration planning engages your team to think through the ways you will work together.  The attached chart gives a high-level overview of the process for creating a Collaboration Plan.

**What will happen during Collaboration Planning?**  In a 90-minute, one-time session with your team, Dr. Betsy Rolland, ICTR Director of Team Science will lead you through a short training on the principles of collaboration, followed by a guided exercise to **craft a plan specific to your pilot project** addressing key challenges of team science.  The end result of this session will be a short, 2-3 page Collaboration Plan.

Your Collaboration Plan may be useful in the following ways:

- To guide your team’s work for this ICTR pilot award
- As a template for writing a multi-PI plan
- To address review criteria specific to team-science  in future grant applications
- Provides practice for trainees and early-stage investigators who will soon be running their own teams.

While this exercise is optional, we highly recommend you take advantage of this opportunity to get your project off to its best-possible start.

**How do you accept or decline this invitation?**  Simply reply to this email to let me know, and we will help to schedule your session with Betsy Rolland.

Please respond to this email at your earliest convenience.  We welcome your questions!

**Appendix C**

**Survey Questions Distributed to Collaboration Planning Participants**

1. Name
2. Please indicate how valuable you found the collaboration planning meeting overall for your team’s early development.
   1. Very valuable
   2. Somewhat valuable
   3. Not particularly valuable
   4. Not valuable at all.
3. Please indicate: a) which 2-3 sections of the collaboration planning template you anticipate using the most in your team's early development, and b) which 2-3 sections you anticipate using the least. (Please choose 2-3 under each heading.)
   1. Rationale for Team Approach & Configuration
   2. Collaboration Readiness
   3. Technological Readiness
   4. Team Functioning
   5. Communication & Coordination
   6. Leadership, Management, & Administration
   7. Conflict Prevention & Management
   8. Training
   9. Quality Improvement Activities
   10. Budget & Resource Allocation
4. Based on conversations with your team thus far, what collaboration issue(s) do you think will be the most problematic for your team? [Open-ended text box]
5. What suggestions do you have for ICTR's Team Science Director (Betsy Rolland) for improving the collaboration planning meetings with teams? [Open-ended text box]
6. If you have any additional questions about collaboration planning that the meeting did not address, please write them here. [Open-ended text box]

**Appendix D**

**Collaboration Planning Worksheet (v3, November 22, 2019)**

**Adapted from *How to Write a Collaboration Plan*, Hall, Crowston, and Vogel [4, 5]**

**[NB: “Resources” are proposed additional materials or referrals that can be given to participants after the session if they have questions or would like additional guidance. These are under preparation.]**

1. Rationale for Team Approach and Configuration
   1. Why are you doing this work as a team?
   2. Who is on the team and what skill set do they bring?
   3. On a more task-based level, who will do what on your team and in collaboration with whom?
   4. How will those roles be communicated across the group, as well as to new team members?
   5. Are there skill sets that are missing from this team?
      1. How will you adjust for those missing skills or fill those holes?
   6. Are there people on the team who maybe don’t need to be?
   7. Often in a team, members contribute different amounts at different periods of time. Will some members’ effort vary across the life of the project?
2. Collaboration Readiness
   1. For members who are new to the team or teams that are new to working together, how will you support the development of trusting relationships? [Resource: one-pager on developing trusting relationships]
   2. How will you identify different work styles among members and help collaborators manage those differences? *[Resource: assessing work styles]*
   3. Do you have early-career researchers on the team that may need guidance on explaining/annotating their role on this project for their promotion package? *[Resource: guidance for APT chairs, committees, applicants]*
3. Technological Readiness
   1. What communication technologies (WebEx, email) will you use to work together?
   2. What coordination technologies (shared calendar, Box, shared drive, project management tools) will you use to work together?
   3. What data management and analysis tools will you use to work together?
      1. Do you need any data use agreements with non-UW partners?
   4. If your collaborators are at other institutions, will the UW resources you’re using be accessible to the entire team?
   5. How will you ensure that all of these tools work together? *[Resource: organizing information for collaborative teams]*
   6. How will you document where each type of information or data lives and train everyone on the team so they know what information or data goes into which tool and where to find different kinds of information or data?
4. Team Functioning
   1. How will your team create a shared vision of what success looks like for the project as a whole and the individual components? *[Resource: one-pager on developing and assessing strong team functioning]*
      1. Kick-off meetings
      2. Highlighting these elements at each meeting
   2. How will your team create shared mental models/frameworks of your project’s scientific concepts and shared vocabulary, especially if your team has a number of disciplines represented who may be approaching their work with different models/frameworks?
   3. Does your team need help with facilitation of any of these processes?
5. Communication & Coordination
   1. How will you communicate as a group (email listserv, Google groups)?
   2. How often will your team meet and by what modality (in-person, WebEx, phone)?
   3. Are there sub-teams that will meet? If so, how will the outcomes of those meetings be communicated to the larger group?
   4. How are the results of those meetings and communications documented and stored so they are accessible to the team and for future use?
   5. How will you onboard and offboard team members in terms of communications?
6. Leadership, Management, and Administration
   1. One of the roles for a leader is to provide vision and direction to the team. How will you take the shared vision your team developed and operationalize it into concrete direction for the team?
   2. How will you create a culture that encourages all team members to show leadership? What does that look like in practical terms?
   3. How will the leadership team ensure that all members are encouraged to participate and add their unique perspectives to the conversation?
   4. How will tasks be identified, assigned, tracked, and judged complete?
   5. How will your team hold one another accountable for delivering high-quality work on time and as agreed upon?
   6. Who on your team is responsible for administrative tasks like organizing meetings, taking minutes, backing up data, tracking due dates, etc?
7. Conflict Prevention and Management
   1. Do you anticipate publications or intellectual property arising from this project?
   2. Where will you publish?
   3. If so, what will your authorship or attribution policies be?
   4. What will your data sharing policy be?
   5. What is your process for resolving disputes such as those over resources or deliverables?
      1. Conflicts among staff vs conflicts between PIs
   6. *[Resource: Local conflict resolution resources, including ombudsperson]*
8. Training
   1. Will the project require collaborators to cross-train on methods or tools?
   2. Will team members need training on how to use the communication, coordination, or data systems?
   3. What team-based skills will the team need, beyond what we’ve done today (e.g., conflict resolution, effective communication, information management, leadership)? *[Resource sheet on what’s available in ICTR and UW-wide]*
9. Quality Improvement Activities
   1. How will your team assess if it is functioning well?
      1. What are some of the red flags for a poorly functioning team and signs of a highly functioning team?
   2. How will you build in reflection time at each milestone?
10. Budget & Resource Allocation
    1. How will you allocate resources to support team function?
